# Little things with significant impact: miRNAs in hepatocellular carcinoma

**DOI:** 10.3389/fonc.2023.1191070

**Published:** 2023-05-19

**Authors:** Jiehan Li, Haolin Bao, Ziyue Huang, Zixin Liang, Mei Wang, Ning Lin, Chunjie Ni, Yi Xu

**Affiliations:** ^1^ Department of Hepatopancreatobiliary Surgery, The Second Affiliated Hospital of Harbin Medical University, Harbin, Heilongjiang, China; ^2^ Key Laboratory of Basic Pharmacology of Ministry of Education, Zunyi Medical University, Zunyi, Guizhou, China; ^3^ Key Laboratory of Functional and Clinical Translational Medicine, Fujian Province University, Xiamen Medical College, Xiamen, Fujian, China; ^4^ Jiangsu Province Engineering Research Center of Tumor Targeted Nano Diagnostic and Therapeutic Materials, Yancheng Teachers University, Yancheng, Jiangsu, China; ^5^ State Key Laboratory of Chemical Oncogenomics, Key Laboratory of Chemical Genomics, Peking University Shenzhen Graduate School, Shenzhen, China; ^6^ Key Laboratory of Biomarkers and In Vitro Diagnosis Translation of Zhejiang Province, Hangzhou Medical College, Hangzhou, Zhejiang, China; ^7^ Key Laboratory of Gastrointestinal Cancer (Fujian Medical University), Ministry of Education, School of Basic Medical Sciences, Fujian Medical University, Fuzhou, Fujian, China; ^8^ Anhui Province Key Laboratory of Translational Cancer Research, Bengbu Medical College, Bengbu, Anhui, China; ^9^ Key Laboratory of Intelligent Pharmacy and Individualized Therapy of Huzhou, Department of Pharmacy, Changxing People’s Hospital, Changxing, Zhejiang, China; ^10^ Department of Pathology, Li Ka Shing Faculty of Medicine, The University of Hong Kong, Hong Kong, Hong Kong SAR, China

**Keywords:** hepatocellular carcinoma, microRNA, tumor microenvironment, autophagy, ferroptosis, metabolic reprogramming

## Abstract

Hepatocellular carcinoma (HCC) has developed into one of the most lethal, aggressive, and malignant cancers worldwide. Although HCC treatment has improved in recent years, the incidence and lethality of HCC continue to increase yearly. Therefore, an in-depth study of the pathogenesis of HCC and the search for more reliable therapeutic targets are crucial to improving the survival quality of HCC patients. Currently, miRNAs have become one of the hotspots in life science research, which are widely present in living organisms and are non-coding RNAs involved in regulating gene expression. MiRNAs exert their biological roles by suppressing the expression of downstream genes and are engaged in various HCC-related processes, including proliferation, apoptosis, invasion, and metastasis. In addition, the expression status of miRNAs is related to the drug resistance mechanism of HCC, which has important implications for the systemic treatment of HCC. This paper reviews the regulatory role of miRNAs in the pathogenesis of HCC and the clinical applications of miRNAs in HCC in recent years.

## 1 Introduction

Hepatocellular carcinoma (HCC) is the main manifestation of primary liver cancer, accounting for 75% to 85% of primary liver cancers and the second leading cause of malignancy-related deaths worldwide (1). Owing to the difficulty of early diagnosis, insidious onset, and ease of recurrence and metastasis of HCC, most patients have proceeded to intermediate and advanced stages by the time they are diagnosed (2). Although treatment techniques such as radiofrequency ablation, surgical resection, and liver transplantation have been continuously advanced in the past decades (3), and certain improvements have been achieved in the treatment of HCC, the 5-year survival rate of HCC patients is still less than 20% because of the high recurrence and metastasis rates after HCC surgery (4). Standard treatment options for intermediate to advanced HCC include sorafenib, doxorubicin, platinum anticancer agents, 5-fluorouracil, and gemcitabine (3). However, because many HCC patients eventually develop resistance to these chemotherapeutic agents, combined with the complexity of the resistance mechanisms in HCC, this leads to poorer prognosis or clinical outcomes for patients (5). Drug resistance has recently become a big problem for HCC treatment. The study of new mechanisms of HCC resistance, resistance markers, and new targets for reversing resistance is the key to improving the efficacy of HCC treatment. Therefore, in-depth studies on the underlying pathogenesis of HCC are urgently needed to develop more effective therapeutic modalities for HCC patients.

MicroRNA (miRNA) is a class of short-stranded non-coding single-stranded small molecule RNAs ranging in length from 19 to 25 nucleotides (6). They are complementarily paired with mRNAs through the principle of base pairing and usually target the binding site on the 3’UTR of mRNAs and carry RISC (including Ago protein and glycine-tryptophan 182) to play the following three roles: transcriptional repression, mRNA cleavage, and mRNA degradation, with transcriptional repression being the most dominant (7, 8). Furthermore, miRNAs are widely present in eukaryotic organisms, control the transcription and translation of many genes in plants and animals, and are among the most important regulators of gene expression (8). It has been reported that miRNAs account for only 1%-3% of the human genome, but they regulate about 33% of human coding protein genes (6). By targeting and inhibiting downstream mRNAs, miRNAs are essential molecules in regulating cellular functions, participating in various processes such as cell proliferation, differentiation, apoptosis, organ development, and maintenance of organ physiology *in vivo* (9).

Viral hepatitis is a common disease worldwide. In HCC, about 85% have hepatitis B virus (HBV) infection, and about 70% are caused by the progression of hepatitis B cirrhosis, but the exact mechanism by which this infection promotes cancer development is not fully understood. Recent studies have shown that miRNAs have an essential role in the development and progression of viral hepatitis, especially in hepatocellular carcinogenesis. For example, HBV can target miR-340-5p to regulate ATF7/HSPA1B-mediated proliferation and apoptosis and promote the progression of HCC (10). MiR-802 promotes HBV DNA replication and HbsAg and HbeAg expression by regulating SMARCE1 expression in HCC cells, which promotes HBV DNA replication and HbsAg and HbeAg expression (11). HBV infection can induce the development of hepatocellular carcinoma by suppressing miR-122 expression and inducing the expression of miR-122 target gene APOBEC2, promoting the growth of HCC cells (12). As with chronic viral hepatitis B, abnormal expression of miRNAs is also present in patients with hepatitis B cirrhosis. Studies have shown that dysregulated miRNAs regulate multiple processes, such as proliferation, apoptosis, invasion, and metastasis of HCC cells, and are closely related to the development and progression of HCC (9, 13). Therefore, an in-depth study of the molecular mechanisms of miRNAs in HCC is of great importance for the prevention and treatment of HCC.

The tumor microenvironment (TME) is the surrounding microenvironment in which tumor cells exist, consisting of stromal cells such as immune cells, tumor-associated fibroblasts, endothelial cells, etc., and extracellular components such as extracellular vesicles, cytokines, and blood vessels, etc. The TME differs from the normal internal environment, characterized by hypoxia, low PH, and inflammatory responses. To support tumor cell development, invasion, and metastasis, tumor cells interact with the tumor microenvironment components to modify the tumor phenotype and establish a growth-supportive milieu. Here, we summarize the recent interactions between miRNAs and TME, the mechanisms by which miRNAs are involved in regulating regulated cell death, mitochondrial function, and metabolic reprogramming and thus influencing HCC progression, thus providing new insights into miRNAs as potential therapeutic targets for HCC.

## 2 Non-cellular components of the tumor microenvironment

### 2.1 Exosome

In 1987, Nelson et al. (14) identified exosomes *in vitro* cultures of reticulocytes from sheep and considered them as a waste product of cells. In 2007, Valadi et al. (15) first confirmed that exosomes contain mRNA and miRNA, called “exosomal shuttle RNA “, which can be transferred to other cells to mediate intercellular communication. With the development of research methods and techniques, researchers discovered that exosomes are model vesicles with a lipid bilayer structure that can carry active substances such as non-coding RNAs, proteins, and lipids, which can be delivered to other target cells through autocrine and paracrine delivery, and together participate in intercellular information exchange in the TME (16). In the last decade, researchers have conducted numerous studies to clarify the exact role of different active substances in exosomes, especially miRNAs. Studies have shown that Exosome-miRNAs (Exo-miRNAs) are involved in several processes related to HCC, such as angiogenesis, immune escape, and metastasis, and are inextricably linked to the biological process of HCC (17).

As a classical tumor suppressor, phosphatase and tensin homolog (PTEN) expression is generally reduced in HCC, and its expression is also regulated by PTEN pseudogene 1 (PTENp1) (18). It has been reported that Exo-miR-21 not only regulates the expression of PTEN, PTENp1, and TETs directly but also increases the methylation level of the PTENp1 promoter by regulating the expression of TETs, which inhibits the expression of PTENp1 and PTEN, promotes the proliferation and migration of HCC cells, and inhibits their apoptosis. Interestingly, miR-21 inhibitors partially attenuated these effects, suggesting that exosomes derived from HCC cells act by transporting highly expressed miR-21 (17). Furthermore, Exo-miR-21 secreted by HCC cells directly targets PTEN and downregulates its expression, resulting in the activation of PDK1/AKT pathway in hepatic stellate cells (HSCs), which ultimately promotes HCC development (19).

However, Exo-miRNA is a “double-edged sword” that can participate in several critical processes such as proliferation, invasion, and metastasis of HCC cells but also inhibit the progression of HCC. In general, Exo-miRNAs secreted by tumor cells can promote tumor progression, but in some exceptional cases, the opposite can be true. For example, HCC cell-derived Exo-miR-125b can directly target SMAD2 and downregulate its expression, further inhibiting TGF-β1-induced EMT and TGF-β1/SMAD pathway, thus suppressing the migration and invasive ability of HCC cells (20). Notably, miR-125b has been reported to play an oncogenic role by downregulating expression in colon cancer and hematopoietic tumors and promoting tumor cell growth and proliferation (21). This also suggests that miR-125b can play a dual role as an oncogene and tumor suppressor for cancer progression. As shown in **Table 1**, we summarize some Exo-miRNAs in HCC and their potential mechanisms (22–28).

**Table 1:** Exosomal miRNAs in HCC.

Exosomal MiRNA	Parental cells	Recipient cells	Expression	Target gene	Potential Mechanism	Ref.
miR-148a-3p	hepatic stellate cells	HCC cells	down	ITGA5	suppressing PI3K/AKT pathway	(22)
miR-92a-3p	high-metastatic HCC cells	low-metastatic HCC cells	up	PTEN	activating Akt/Snail pathway	(23)
miR-199a-3p	mesenchymal stem cells	HCC cells	up	mTOR	inhibiting mTOR pathway	(24)
miR-20a-5p	cancer-associated fibroblast	HCC cells	up	LIMA1	activating Wnt/β-catenin pathway	(25)
miR-4800-3p	HCC cells		up	STK25	activating Hippo pathway	(26)
miR-628-5p	M1 macrophages	HCC cells	up	METTL14	inhibiting m6A modification of circFUT8	(27)
miR-21-5p	HCC cells	THP-1 cells	up	RhoB	regulating MAPK pathway and inducing macrophage M2 polarization	(28)

ITGA5, integrin Subunit Alpha 5; LIMA1, LIM domain and actin binding 1; STK25, serine/threonine protein kinase 25; METTL14, methyltransferase-like 14; THP-1, human monocyte-derived leukemia; RhoB, Ras homolog family member B.

### 2.2 Vessel

#### 2.2.1 Endothelium-dependent angiogenesis

Normally, neoangiogenesis is regulated in the body by a combination of pro- and anti-angiogenic factors that regulate vascular homeostasis. However, in the malignant setting, proliferating tumors disrupt the angiogenic balance and promote angiogenesis (29). Tumor angiogenesis refers to the degradation of extravascular stroma and basement membrane by vascular endothelial cells in differentiated vessels in the presence of pro-angiogenic signaling molecules such as VEGF and FGF. The process of endothelial cell proliferation and migration to re-form the vascular network (30). Studies have shown that the dysregulation of miRNAs is directly related to the angiogenic process in HCC. For example, miR-130b-3p expression was significantly increased in HCC and downregulated its expression by directly targeting HOXA5, which further activated the PI3K/AKT/mTOR pathway, thereby stimulating HCC cells to induce capillary tube formation, endothelial cell migration, and proliferation. Notably, Sp1 can also further promote HCC angiogenesis by binding to the miR-130b-3p-promoter and thus regulating miR-130b-3p expression (31).

In addition, miRNAs can be released into recipient cells via exosomes secreted by HCC cells, thereby mediating intercellular communication in the TME. A significant correlation between serum miR-210 levels and the number of microvessels in HCC tissues has been reported in HCC patients. Mechanistically, miR-210 was transferred to endothelial cells via exosomes secreted by HCC cells, thereby further promoting *in vitro* tubulogenesis and *in vivo* angiogenesis in endothelial cells by targeting SMAD4 and STAT6 and directly inhibiting their expression. Interestingly, this study demonstrated that miRNAs could serve as a new class of mediators of the interaction between HCC cells and endothelial cells (32). Xiao et al. (33) confirmed that miR-32-5p can be transferred from drug-resistant cells to sensitive cells via exosomes and induce HCC-sensitive cells by suppressing PTEN activation of the PI3K/AKT pathway and ultimately promoting angiogenesis induction of multidrug resistance in HCC-sensitive cells. In this study, miRNAs emerged as regulators of HCC angiogenesis and the transformation of sensitive cell lines into multidrug-resistant cell lines, providing us with new ideas for future studies to improve drug sensitivity in treating HCC. We summarized some miRNAs that promote HCC angiogenesis based on recent studies, as shown in **Table 2** (19, 31–38).

**Table 2:** Dysregulated miRNAs promote angiogenesis in HCC.

MiRNA	Source	Expression	Target gene	Mechanism	Ref.
miR-130b-3p	tissue	up	HOXA5	activating PI3K/AKT/mTOR pathway	(31)
miR-210	tumor-derived exosome	up	SMAD4, STAT6	promoting endothelial cell tubulogenesis *in vitro* and HCC *in vivo* angiogenesis	(32)
miR-32-5p	tissue, exosome	up	PTEN	activating PI3K/AKT pathway	(33)
miRNA-21	tumor-derived exosome	up	PTEN	converting HSCs to CAFs and secreting angiogenic cytokines	(19)
miR-200b-3p	tumor-derived exosome	down	endothelial ERG	enhancing endothelial ERG expression	(34)
miR-1290	serum-derived exosome	up	SMEK1	alleviating SMEK1’s suppression of VEGFR2 phosphorylation	(35)
miR-325-3p	tissue, cell	down	CXCL17	secreting angiogenic cytokines	(36)
miR-1178-3p	tissue	down	TBL1XR1	activating PI3K/AKT pathway	(37)
miR-584-5p	cell, EV	up	PCK1	activating NRF2 pathway	(38)

HOXA5, Homeobox A5; STAT6, signal transducer and activator of transcription 6; ERG, erythroblast transformation-specific (ETS)-related gene; SMEK1, suppressor of MEK1; CXCL17, C-X-C motif chemokine ligand 17; TBL1XR1, transducin (beta)-like 1 X-linked receptor 1; PCK1, phosphoenolpyruvate carboxykinase 1; NRF2, nuclear factor E2-related factor 2.

#### 2.2.2 Vascular mimicry

Maniotis et al. (39) proposed a novel way of blood supply to tumors in their study of malignant melanoma of the human uvea. This approach consists of tumor cells deforming themselves and interacting with the extracellular matrix to form a tubular meshwork that can deliver blood and mimic the structure of the vascular wall, allowing the tumor to obtain blood supply, known as vascular mimicry (VM) (39). The proposal of VM challenges the conventional notion that endothelium-dependent vascularity is the only way of tumor microcirculation and enriches the existing theory of tumor blood supply. It also means that when angiogenesis is inhibited with vasopressors, cancer cells have a second option to obtain nutrients and oxygen, thus becoming “cancer cells that cannot die of hunger”. As research progresses, this vascular mimetic system has been shown to exist in a variety of tumors, including HCC (40), breast cancer (41), and gastric cancer (42), posing a significant threat to patient survival. To date, researchers have identified several proteins that may affect VM in aggressive HCC, including HIF-1α, MMP2, and Twist-1 (43, 44), and miRNAs play a crucial part in the VM process by regulating their expression. It has been revealed that miR-138-5p functions as a tumor suppressor in HCC and suppresses VM by binding to the 3′UTR of HIF-1α mRNA and further downregulating the expression of HIF-1α and VEGFA (43). Twist-1 is a transcription factor having the structure of a helix-loop-helix. Studies have shown that Twist-1 can directly bind to the promoter region of miR-27a-3p and regulate its expression to induce invasive cell malignant behavior and VM formation. In addition, miR-27a-3 is downregulated in Twist-1 high-expressing HCC cells. It can regulate the VM process in HCC by inhibiting the expression of VE-cadherin (VE-Cad) and MMP2, thus regulating EMT inhibition (44). Yang et al. (45) revealed that cancer-associated fibroblasts paracrine TGF-β and SDF1 enhanced the expression of VE-Cad, MMP2, and LAMC2 in HCC cells through TGF-βR1 and CXCR4, thus promoting VM formation. Interestingly, miR-101 can inhibit VM formation by targeting multiple molecules in CAFs and HCC cells. Mechanistically, miR-101 targets TGF-βR1 and Smad2 in HCC cells to attenuate TGF-β signaling while eliminating SDF1 signaling by inhibiting SDF1 expression in CAFs and suppressing VE-Cad expression in HCC cells, thereby blocking VM formation induced by CAFs (45). In conclusion, miRNAs can act as oncogenic factors to inhibit VM formation and hold promise as anti-VM molecules in HCC. Unfortunately, little is known about the importance of miRNAs in the VM process in HCC cells. The specific mechanism of miRNAs in regulating VM formation has not been adequately described in the current study.

#### 2.2.3 Vascular permeability

**Figure 1:**
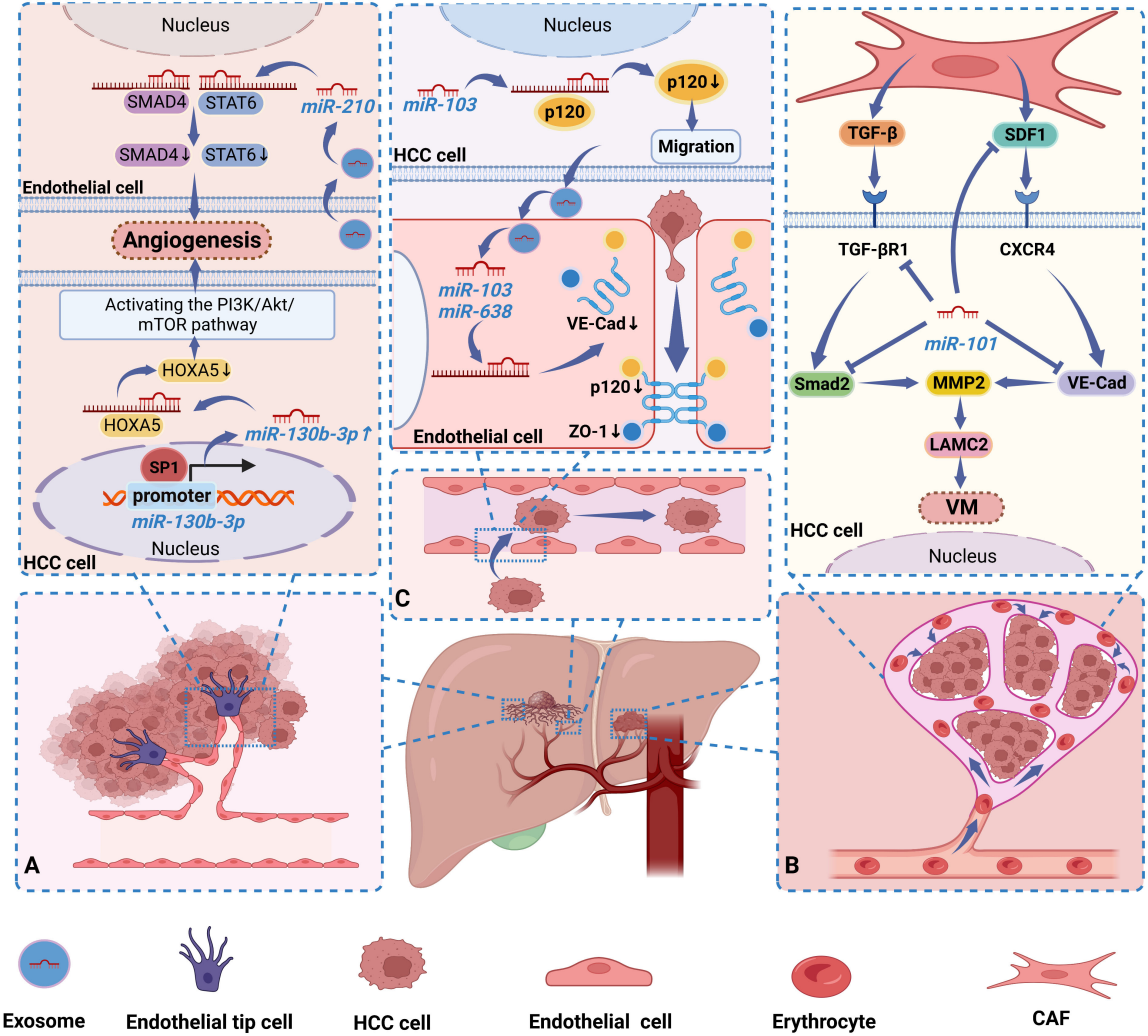
Dysregulated miRNAs regulate HCC progression by affecting blood vessels in tumors in 3 ways. **(A)** Sp1 maintains a high miR-130b-3p expression state by binding to the miR-130b-3p-promoter, while miR-130b-3p activates the PI3K/AKT/mTOR pathway by inhibiting HOXA5 expression, thereby stimulating HCC cells to induce angiogenesis. Exo-miR-210 is secreted by HCC cells into endothelial cells to promote angiogenesis by inhibiting SMAD4 and STAT6 expression. **(B)** MiR-101 can inhibit VM formation by targeting multiple molecules in CAFs and HCC cells and downregulating their expression. **(C)** Exo-miR-638 is delivered by HCC cells into endothelial cells and down-regulated their expression by targeting VE-Cad and ZO-1. Similarly, Exo-miR-103 was secreted into endothelial cells by HCC cells and suppressed the expression of VE-Cad, p120, and ZO-1. In addition, miR-103 in HCC cells also inhibited the expression of p120. They confer a strong metastatic potential to HCC cells by increasing the permeability of blood vessels.

In the early stage of metastatic growth, cancer cells will induce the formation of a local TME suitable for metastasis, that is, the formation of a premetastatic niche (PMN), which is a “fertile ground” for the growth of tumor cells. Cancer-induced vascular permeability is a critical step in the initiation of PMN (46). The junctions between endothelial cells, consisting of adherens junctions and tight junctions, are essential for maintaining the functional integrity of the vasculature and play a crucial role in vascular permeability. Extensive studies over the last decade have identified several molecular components of tight and adherent junctions, including VE-Cad, catenins (p120-catenin and β-catenin), and zonula occludens 1 (ZO-1) (47). Downregulation or deletion of these proteins would degrade endothelial junction integrity and enhance vascular permeability, hence promoting cancer cell metastasis. It has been shown that miRNAs can act as oncogenic factors in HCC by regulating the integrity of endothelial junctions, thereby promoting HCC metastasis. Exo- miR-638, secreted from highly intrahepatic metastatic cells, was reported to promote increased vascular permeability by targeting VE-Cad and ZO-1 in endothelial cells and downregulating their expression, which in turn initiates PMN (46). In another study, miR-103 secreted by HCC cells could be delivered to endothelial cells via exosomes and then directly inhibit the expression of the connexins VE-Cad, p120 and ZO-1, thereby weakening the integrity of endothelial junctions and increasing vascular permeability. Interestingly, p120 can stabilize E-Cad at the cell membrane by blocking E-Cad endocytosis and degradation via binding to E-Cad. At the same time, miR-103 in HCC cells can also promote tumor cell migration by inhibiting p120 (48). This suggests that exogenous miR-103 is delivered to endothelial cells via exosomes in a paracrine manner and lowers the endothelial barrier, and endogenous miR-103 in HCC cells enhances cell migration, which together confers a strong metastatic potential to HCC cells (48). Thus, miRNAs in HCC cells play a crucial role in regulating tumor vascular integrity and cancer metastasis. In **Figure 1**, we illustrate three ways in which miRNAs in HCC affect the vasculature in tumors (31, 32, 45, 46, 48).

### 2.3 Hypoxia and acidic microenvironment

It is well known that TME can serve as a “soil” for tumor cells to survive, providing the basis for tumorigenesis and development. Unlike the normal internal environment of the human body, TME is characterized by tissue hypoxia and acidosis (49, 50). Studies have shown that the hypoxic and acidic microenvironment in HCC induces the expression of miRNAs, which in turn plays an essential role in tumor progression. For example, miR-1307-3p can act as a novel hypoxia response factor and is subject to transcriptional regulation by HIF-1α. Importantly, DAB2IP acts as a direct target of miR-1307-3p, and hypoxia-driven miR-1307-3p promotes the proliferation and invasion of HCC cells by inhibiting DAB2IP. Furthermore, in a hypoxic microenvironment, HIF-1α indirectly inhibits DAB2IP levels through upregulation of miR-1307-3p, while downregulated DAB2IP further maintains HIF-1α levels by activating AKT/mTOR signaling, forming a feedback loop in HCC cells (51). Tian et al. (52) found that in HCC cultured under acidic conditions cells, increased HIF-1α and HIF-2α could directly bind to the HRE sites in the promoters of miR-21 and miR-10b and upregulate miR-21 and miR-10b expression. Further studies confirmed that the acidic microenvironment induced the expression of Exo-miR-21 and Exo-miR-10b through HIF-1α or HIF-2α and promoted the proliferation, migration, and invasion of HCC cells. Thus, the hypoxic and acidic microenvironment in HCC is crucial in promoting HCC proliferation and metastasis by inducing altered expression of miRNAs.

## 3 Cellular components of the tumor microenvironment

### 3.1 Cancer stem cell

Cancer stem cells (CSCs) are a functional subpopulation of cells with stem cell characteristics in tumor tissues that have the potential to be able to self-renew and multi-directionally differentiate in an undifferentiated state. There is no doubt that CSCs can proliferate indefinitely and have the potential for high invasiveness and metastasis, and are associated with local recurrence or distant metastasis in a variety of tumors, including HCC (53). In recent years, many scholars have conducted numerous studies on how miRNAs regulate the biological behavior of liver cancer stem cells (LCSCs) and found that dysregulation of specific miRNAs is a key regulator of CSC subpopulations and contributes to the regulation of the expansion, self-renewal, and drug resistance of LCSCs. For example, aberrantly expressed miRNAs can downregulate the expression of target genes and thus regulate cancer-related signaling pathways involved in the maintenance of the phenotype of LCSCs. Overexpression of miR-302a/d in HCC cells was reported to significantly increase the expression ensemble of CSC markers and the efficiency of spheroid formation, suggesting that miR-302a/d may suppress the stemness of HCC cells. Mechanistically, miR-302a/d acts as an essential repressor of LCSCs by inhibiting the target gene E2F7 and its downstream AKT/β-catenin/CCND1 pathway, thereby negatively regulating the self-renewal capacity and cell cycle entry of LCSCs (54). In another study, miR-1305 directly targeted UBE2T to suppress the activation of the AKT signaling and ultimately suppressed spheroid formation, colony formation and proliferation, and tumorigenicity of LCSCs. This suggests that miR-1305 inhibits the expansion and self-renewal of LCSCs and could play an essential role as a potential therapeutic target in the treatment of HCC (55). FOXO1, as a tumor suppressor, interacts with β-catenin in the cytoplasm and reduces β-catenin nuclear translocation of β-catenin, which is a crucial step in the activation of the Wnt/β-catenin pathway (56). Further studies revealed that HBX-induced miR-5188, a down-regulated miRNA in HCC, could directly target FOXO1 and down-regulate its expression, stimulating nuclear translocation of β-catenin, promoting Wnt signaling activation and downstream c-Jun expression, and ultimately promoting the stemness of LCSCs. Interestingly, miR-5188 is regulated by c-Jun, a downstream effector of the wnt/β-catenin pathway, whose transcription increases miR-5188 expression and forms a positive feedback loop (56). Furthermore, CSCs are closely associated with tumor drug resistance. Unfortunately, there are fewer reports on miRNAs regulating drug resistance in LCSCs. In **Table 3**, we summarized the regulatory roles of some miRNAs in LCSCs (54–64).

**Table 3:** Dysregulated miRNAs in LCSCs.

MiRNA	Expression	Process	CSCs marker	Pathway	Ref.
miR-302a/d	down	self-renewal	CD133, EpCAM	miR-302a/d/E2F7AKT/β-catenin/CCND1	(54)
miR-1305	down	expansion, self-renewal	CD133, CD13	miR-1305/UBE2T/AKT	(55)
miR-5188	up	expansion	CD133, CD44	HBX/miR-5188/FOXQ1/Wnt/β-catenin	(56)
miR-6875-3p	up	expansion	CD133, EpCAM	miR-6875-3p/BTG2/FAK/AKT	(57)
miR-448	down	self-renewal	CD133, CD44	miR-448/MAGEA6/AMPK	(58)
miR-2392	up	expansion	CD133, EpCAM	miR-2392/JAG2/Notch	(59)
miR-4319	down	expansion	CD44, EPCAM	miR-4319/FOXQ1	(60)
miR-186	down	expansion, self-renewal	CD133, CD90, CD24, EpCAM	miR-186/PTPN11	(61)
miR-206	down	expansion, self-renewal	CD133, EpCAM	miR-206/EGFR	(62)
miR-365	down	expansion, self-renewal	CD133, EpCAM	miR-365/RAC1	(63)
miR-219	up	expansion, self-renewal	CD133, EpCAM	miR-219/E-cadherin	(64)

E2F7, E2F transcription factor 7; UBE2T, ubiquitin-conjugating enzyme E2T; HBX, HBV X protein; FOXO1, forkhead box protein O1; BTG2, B-cell translocation gene 2; MAGEA6, melanoma-associated antigen 6; EGFR, epidermal growth factor receptor; RAC1, Ras-related C3 botulinum toxin substrate 1.

### 3.2 Tumor-associated macrophage

#### 3.2.1 M1and M2

**Figure 2:**
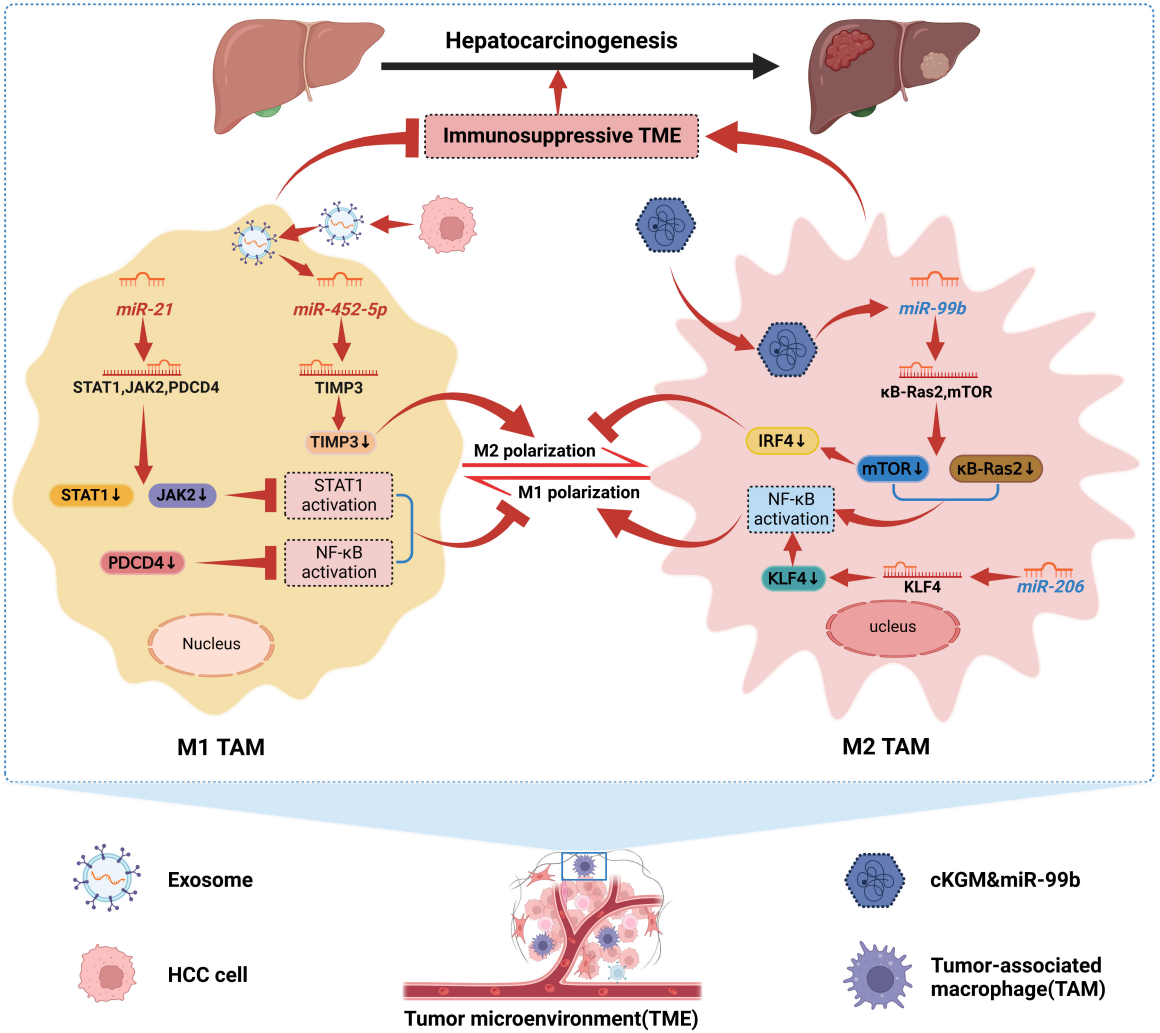
Mechanisms by which miRNAs in HCC drive the interconversion between M1-like and M2-like TAMs. We can conclude that miRNAs dynamically promote the conversion between M1-like and M2-like TAMs. MiR-21 can inhibit STAT1 and NF-κB activation, and Exo-miR-452-5p can target TIMP3 and down-regulate its expression. Both contribute to HCC cellular immune escape by inhibiting M1 polarization of TAMs and inducing M2 polarization of TAMs, respectively, thereby promoting HCC progression. In addition, miR-99b delivered to TAMs and miR-206 in TAMs induced M1 polarization of TAMs by enhancing NF-κB activity; meanwhile, miR-99b also inhibited M2 polarization of TAMs. Together, they confer TAMs immune surveillance and the ability to kill tumor cells to suppress HCC.

With the increasingly clear relationship between tumor-associated macrophages (TAMs) and malignant tumors, TAMs have become a hot spot for scholars to focus on. TAMs are traditionally differentiated into two distinct subtypes, classically activated (M1), which has a role in killing tumor cells and resisting pathogen invasion, and alternatively activated (M2), which promotes tumor cell genesis, invasion, and metastasis (65). There is growing evidence that both M1-like and M2-like TAM are highly plastic. M1 and M2 are the two extremes of dynamically changing TAM, and that dynamic changes in TAM phenotype also occur during tumorigenesis, invasion, and metastasis (66). For example, in the early stages of HCC formation, most TAMs retain the general “angelic” face of M1, which attacks and clears tumor cells. As the tumor progresses, M1-like TAMs will gradually polarize to M2-like TAMs and finally become “demons”, promoting immune escape, invasion, and metastasis of HCC cells, thus driving the progression of HCC (67). Interestingly, dysregulated miRNAs have been shown to induce TAMs to M2-like polarization and promote HCC progression. HCC cells stimulated miR-21 expression in TAMs and were reported to inhibit STAT1 and NF-κB activation by downregulating STAT1, JAK2, and PDCD4 expression, inhibiting the M1 polarization of TAMs. In addition, STAT1 and NF-κB were activated in TAMs to drive M1 polarization in the presence of miR-21 depletion in TAMs (68). Hu et al. (69) found that Exo-miR-452-5p secreted by HCC cells could target TIMP3 to induce M2 polarization in TAMs and was further enhanced by overexpression of miR-452 -5p was further enhanced after overexpression. Furthermore, this study confirmed that Exo-miR-452-5p accelerated HCC cell invasion and migration as well as tumorigenesis, demonstrating that Exo-miR-452-5p induced M2-like TAM polarization to accelerate HCC progression by inducing M2-like TAM. Notably, since TAM is phenotypically plastic, current immunotherapeutic strategies mainly target TAM by depleting the amount of M2-like TAM and promoting M2-like to M1-like TAM conversion. miRNAs can precisely regulate this immune response process and thus influence HCC progression, and are a potential immunotherapeutic approach for HCC. For example, miR-144/miR-451a, a tumor suppressor in HCC cells, can target HGF and MIF in HCC cells and induce M1 polarization and anti-tumor activity of TAMs via paracrine pathway (70). Liu et al. (71) demonstrated that miR-206 promoted CCL2 production by directly targeting the KLF4-NF-κB axis, which drove M1 polarization and promoted CD8 T cell recruitment in Kupffer cells (KCs), and prevented HCC altogether. This suggests that miR-206 can enhance immune surveillance and represents a novel immunotherapeutic treatment for HCC. After miR-99b was reported to be targeted for delivery to TAMs, it reprogrammed the anti-tumor immune microenvironment by mediating TAM remodeling to an anti-tumor phenotype, thereby inhibiting HCC progression (72). On the one hand, miR-99b delivered to TAMs enhances NF-κB activity by targeting κB-Ras2 and mTOR, respectively, promoting M1 polarization. On the other hand, high expression of miR-99b in TAMs inhibited M2-like TAM polarization by suppressing mTOR/IRF4 expression. Interestingly, miR-99b overexpressed M1-like TAMs have stronger phagocytosis and antigen presentation, and whether this is associated with the involvement of NF-κB or mTOR signaling remains to be further investigated (72). Therefore, exploring the mechanism of miRNAs’ role in TAM remodeling will likely contribute to the therapeutic strategy of HCC. Although preliminary findings are available, clinical applications need to be explored in depth. In **Figure 2**, we show the mechanism by which miRNAs in HCC drive the interconversion between M1-like and M2-like TAM (68, 69, 71, 72).

#### 3.2.2 TAMs and HCC Cells: Intercellular Crosstalk

It is well-established that the interaction between TAMs and tumor cells plays an essential role in driving tumor progression. In particular, exosome-delivered miRNAs may represent a crosstalk mode between TAMs and HCC cells with high efficiency and specificity (73). Liu et al. (74) confirmed that exosomes derived from TAMs mediated the transfer of miR-92a-2-5p to HCC cells and increased the invasive capacity of HCC cells, while inhibitors of miR-92a-2-5p may inhibit the function of TAMs on HCC progression. Mechanistically, Exo-miR-92a-2-5p could downregulate AR expression and alter PHLPP/p-AKT/β-catenin signaling to increase HCC cell invasion. Another study revealed that endoplasmic reticulum-stressed HCC cells can deliver miR-23a-3p-rich exosomes to TAMs and that these Exo-miR-23a-3p can regulate the immune function of TAMs to promote HCC progression (75). Further studies on the mechanism showed that miR-23a-3p delivered to TAMs by HCC cells activated the PI3K-AKT pathway by inhibiting PTEN expression, thereby upregulating PD-L1 expression in TAMs and suppressing T-cell function. This implies that miR-23a-3p acts as a critical factor that helps tumor cells to escape from immune surveillance (75). Therefore, interfering with HCC cell-TAM crosstalk may be a viable therapy option for HCC.

### 3.3 Cancer-associated fibroblast

**Figure 3:**
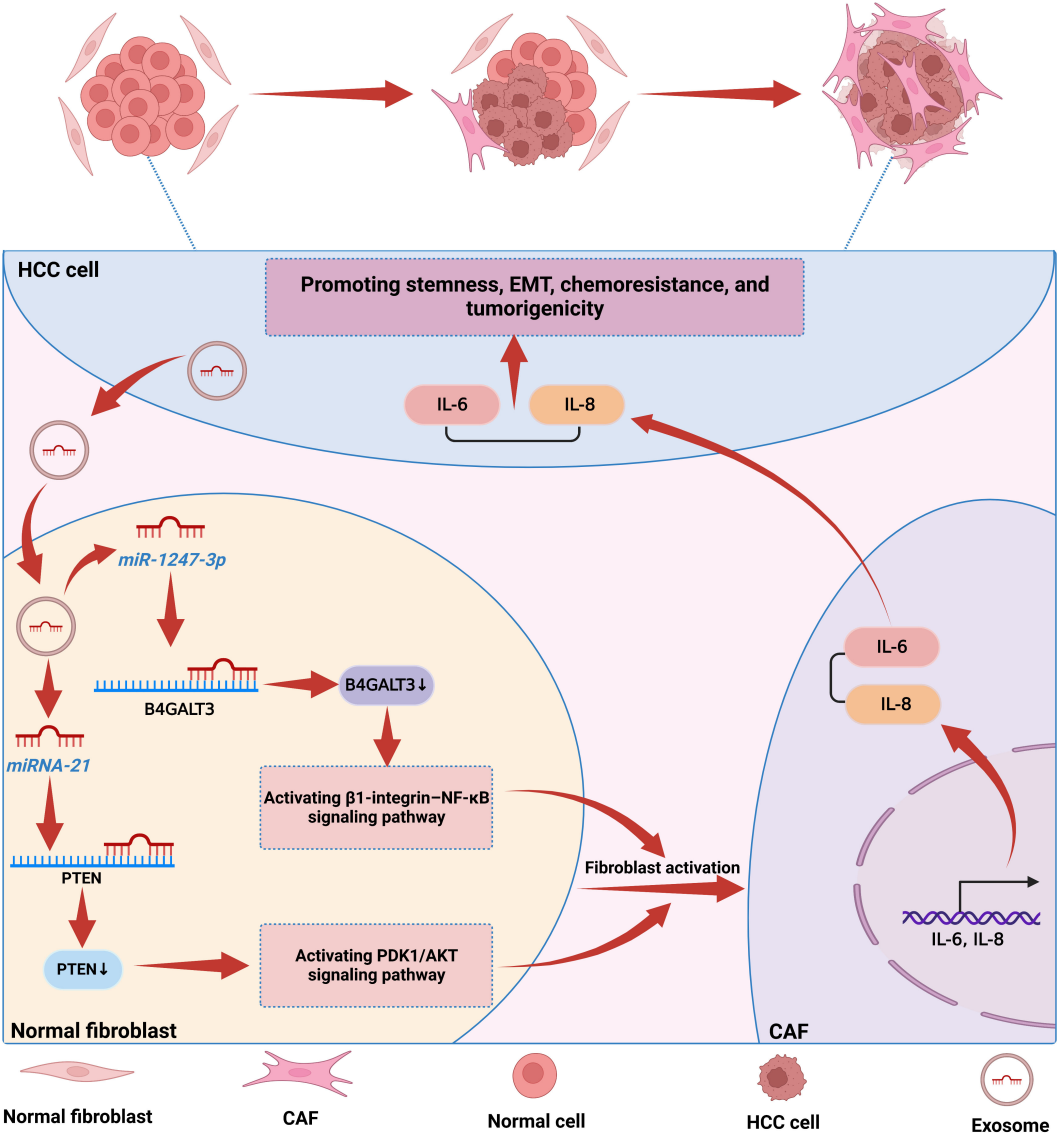
The mechanism by which miRNAs in HCC induce the activation of NFs into CAFs. NFs usually remain resting in normal cells, and when tumors occur *in vivo*, HCC cells transfer miRNAs to NFs *via* paracrine secretion. MiRNAs in NFs induce the conversion of NFs into CAFs by activating cancer-related signaling pathways, and activated fibroblasts further promote the malignant behavior of HCC cells by secreting IL-6 and IL-8.

Cancer-associated fibroblasts (CAFs), an active subpopulation of fibroblasts, are a critical component of the TME. They are the predominant cell type in the tumor stroma and are directly associated with tumor growth, invasion, and metastasis (76). In recent years, the crosstalk between CAFs and HCC cells has been extensively studied (77). It has been shown that CAFs are able to secrete cytokines and immunomodulatory factors, such as miRNAs, which act on tumor cells in a paracrine manner through exosomes, thereby regulating tumor progression (78). In general, CAFs can promote tumorigenesis. However, in some cases, CAFs may also inhibit tumor progression (78). For example, miR-320a is secreted by CAFs and transferred to HCC cells via exosomes. The miR-320a delivered to HCC cells suppresses the activation of the MAPK pathway by directly targeting PBX3, thereby suppressing tumor progression (78). Notably, normal fibroblasts (NFs) usually remain resting, and CAFs are not present in normal humans (79). However, when tumors occur *in vivo*, Exo-miRNAs secreted by HCC cells can be taken up by NFs or hepatic stellate cells (HSCs) and induce the conversion of NFs or HSCs into CAFs (19, 80). More importantly, activated CAFs further promote the growth of HCC by secreting angiogenic cytokines or pro-inflammatory cytokines, thus creating a vicious cycle (19, 80). It has been reported that HCC cells transfer Exo-miR-21 to HSCs in the tumor parenchyma via the paracrine pathway. MiR-21 delivered to HSCs by HCC cells activates the PDK1/AKT pathway by downregulating PTEN expression, thereby converting HSCs into CAFs. Furthermore, CAFs accelerate tumor progression by secreting angiogenic cytokines to promote angiogenesis further (19). Similarly, in another study, miR-1247-3p was shown to transfer to NFs via exosomes secreted by highly metastatic HCC cells and directly target B4GALT3 to activate the β1-integrin-NF-κB pathway, thereby converting NFs to CAFs. Furthermore, CAFs convert NFs to CAFs by increasing IL-6 and IL-8 secretion, thus promoting stemness, EMT, and tumorigenicity in HCC cells (80). Through these studies, we can find that miRNAs play a crucial role in activating NFs or HSCs by HCC cells as mediators of crosstalk between HCC cells and CAFs. Interestingly, miRNAs and CAFs play pro- or anti-tumor roles in the progression of HCC due to the comprehensive source of CAFs and the diversity of secretory functions, reflecting the functional heterogeneity of CAFs. Therefore, in-depth characterization of different CAFs subtypes is necessary before designing new anti-CAFs therapeutic strategies. In **Figure 3**, we show the mechanism by which miRNAs induce the activation of NFs into CAFs and further promote the progression of HCC (19, 80).

## 4 Regulated cell death

### 4.1 Autophagy

Autophagy is a highly conserved catabolic process in eukaryotic cells that degrades damaged intracellular proteins and organelles, mainly through lysosomes. It is essential for maintaining the balanced metabolism of intracellular proteins, the intracellular environment’s stability, and cells’ normal physiological functions (81). Recent studies have revealed that autophagy is closely related to HCC, and the impact of autophagy in HCC is still controversial (82, 83). Most scholars now believe that autophagy can play a dual role in tumor promotion and tumor suppression (84). On the one hand, in the early stages of HCC development, autophagy can induce autophagic cell death to counteract tumor formation. On the other hand, in the middle and late stages of HCC development, autophagy may act as a survival mechanism that enhances the tolerance of HCC cells, thus increasing the malignancy of the tumor (84). Studies have shown that miRNAs influence tumor progression by regulating the autophagic process in HCC cells. For example, miR-135a, which is highly expressed in HCC cells, inhibits autophagy by downregulating the expression level of Atg14, a key component involved in autophagosome formation. In addition, TF/FVII/PAR2 signaling could induce miR-135a expression to inhibit autophagy further. Notably, there is a strong positive correlation between miR-135a levels and malignant behavior in HCC patients, implying that autophagy has a positive role in inhibiting tumor progression (85). However, most studies suggest that autophagy is a mechanism that promotes tumor development and progression. Wei et al. (86) found that miR-513b-5p mimics inhibited the expression of autophagy-related markers LC3-II and beclin1 in HCC cells, which suppressed autophagy in promoting HCC progression by downregulating the expression of PIK3R3, thereby inhibiting cell proliferation, migration, and invasion. Similarly, another study confirmed that miR-490-3p inhibits autophagy in HCC cells by targeting ATG7, suppressing cell proliferation, delaying the cell cycle, and inducing apoptosis (87). Therefore, we conclude that autophagy is crucial for the development of HCC and that miRNAs may be a viable therapeutic target for HCC as an autophagy inhibitory factor.

**Figure 4:**
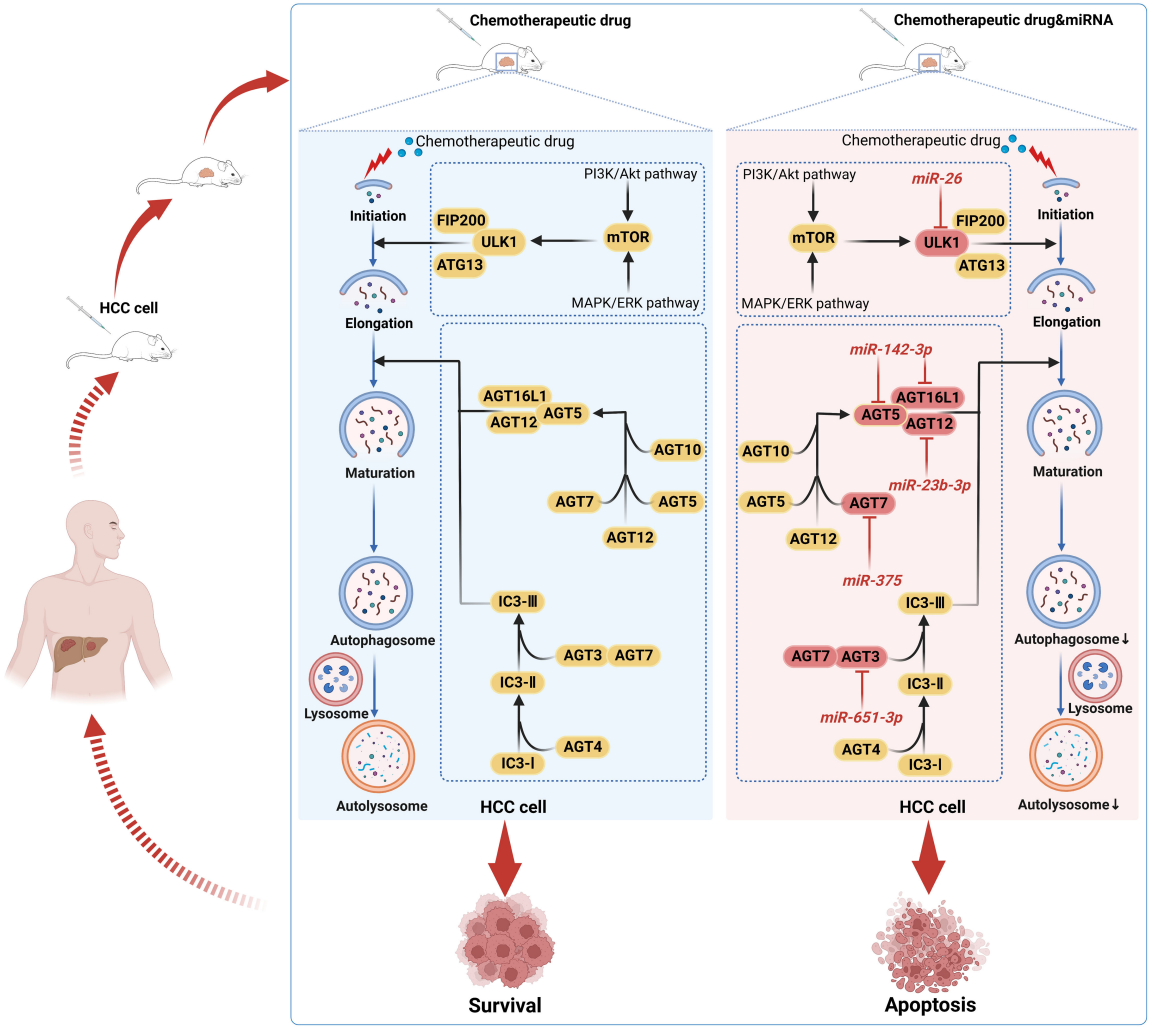
Mechanisms by which chemotherapeutic drugs induce autophagy and miRNAs inhibit autophagy from enhancing the sensitivity of HCC cells to chemotherapeutic drugs. Autophagy may shield HCC cells from the toxic effects of chemotherapeutic drug-induced death, promote HCC survival, and induce drug resistance in a wide range of HCC cells. MiRNAs may act as autophagy inhibitors to reverse the resistance of HCC cells to chemotherapeutic drugs by inhibiting the autophagic process in HCC cells, thereby promoting HCC cell apoptosis.

Indeed, as a cellular defense mechanism, autophagy can reduce the toxicity of chemotherapeutic drugs in HCC cells and minimize the therapeutic effect of drugs on HCC cells, thus inducing chemoresistance in HCC cells (88). By inhibiting autophagy, a rising number of studies have demonstrated that some miRNAs can effectively overcome or reverse chemoresistance in HCC cells. Adriamycin (ADR) is commonly used for transarterial chemoembolization (TACE), but its treatment may be prone to trigger induced autophagy, which leads to cellular chemoresistance (89, 90). Both the chemotherapeutic agent ADR and the autophagy inducer rapamycin have been reported to downregulate miR-26a/b levels in HCC cells. In turn, miR-26a/b overexpression enhanced the sensitivity of HCC cells or implanted tumors to ADR. Further studies revealed that miR-26a/b could promote apoptosis and enhance the sensitivity of HCC to ADR by inhibiting the expression of ULK1, a key promoter of autophagy (91). In another study, miR-26b was demonstrated to enhance the sensitivity of HCC to ADR through USP9X-dependent p53 degradation and regulation of autophagy (92). Interestingly, miR-26b could be involved in regulating miR-26b/ULK1 and miR-26b/USP9X/p53 pathways to enhance ADR sensitivity further. Zhou et al. (93) found that miR-223 suppresses ADR-induced autophagy and reverses chemoresistance to ADR in HCC cells by targeting FOXO3a, which can be used as a potential miRNA-based strategy for autophagy interference. Sorafenib is presently a first-line systemic targeted agent for the treatment of advanced HCC, but its acquired resistance has been a significant obstacle to its clinical application (94). Xu et al. (95) confirmed that miR-541 expression was downregulated in HCC patient tissues, and the combination of overexpressed miR-541 and sorafenib could enhance the sensitivity of sorafenib to HCC cells both *in vitro* and *in vivo* and inhibit the malignant properties of HCC cells. Further studies confirmed that this effect was achieved by miR-541 inhibiting autophagy through downregulating the expression of autophagy-associated gene 2A and Ras-associated protein Rab-1B (95). In addition, miRNAs can be used as sensitizers for cisplatin and oxaliplatin in HCC treatment. For example, miR-651-3p enhances cisplatin cytotoxicity in HCC cells by negatively regulating ATG3-mediated autophagy; miR-125b inhibits EVA1A-mediated autophagy to reverse oxaliplatin resistance in HCC cells (96, 97). Therefore, we conclude that miRNAs in HCC inhibit cellular autophagy and reverse HCC chemoresistance by altering the expression of autophagy-related genes or affecting key links in the autophagic process, providing new therapeutic targets for HCC. In **Figure 4**, we illustrate the mechanism by which some miRNAs enhance the chemosensitivity of HCC by mediating HCC cellular autophagy (91, 96, 98–100).

### 4.2 Ferroptosis

Ferroptosis is a non-apoptotic regulated cell death caused by the iron-dependent accumulation of lipid peroxides (101). It is mainly manifested by abnormally elevated intracellular iron ion levels and a massive collection of reactive oxygen species (ROS), which disrupts the original intracellular oxidation-reduction balance, causing cell membrane damage and, ultimately, cell death (102). Ferroptosis has a dual effect in tumorigenesis, both promoting and suppressing tumor growth, which is dependent on the release of damage-related molecular patterns in the TME and the activation of immune responses induced by ferroptosis damage (103). Studies have shown that miRNAs play an essential role in the progression of HCC by regulating ferroptosis. For example, Exo-miR-142-3p secreted by HBV-HCC cells is transported to M1-like macrophages and promotes ferroptosis of M1-like macrophages by inhibiting the expression of SLC3A2, thereby accelerating the development of HCC (104). MiR-214 can further enhance ferroptosis inducer erastin-induced reduction of GSH and accumulation of ROS and MDA in HCC cells by downregulating the expression of ferroptosis negative regulator ATF4, thus enhancing ferroptosis in HCC cells (105). Studies have demonstrated that sorafenib can induce ferroptosis in HCC cells, resulting in iron toxicity and lipid peroxidation (106). MiR-23a-3p has been reported to be an essential regulator in the development of sorafenib resistance in HCC and can be induced by ETS1 to maintain a high expression status. In addition, miR-23a-3p negatively regulates sorafenib-induced ferroptosis by targeting ACSL4 and downregulating its expression, thereby reducing iron overload and lipid peroxidation, ultimately resulting in sorafenib resistance in HCC cells (107). Interestingly, erastin is currently recognized as the most commonly used inducer of ferroptosis, and its combination with sorafenib may be a viable therapy for HCC., especially in the context of sorafenib resistance. Meanwhile, the interaction between miRNAs and ferroptosis in HCC is still being further explored, while targeting HCC metastasis, energy metabolism, and sorafenib resistance are all directions to be explored in the future.

## 5 Subcellular structure: mitochondrion

Mitochondrion, a critical organelle in most cells to maintain cell survival and homeostasis, is composed of the outer membrane, interstitial space, inner membrane, and mitochondrial matrix (108). The function of mitochondria is closely related to other organelles. It can be involved in various cellular activities such as apoptosis, autophagy, oxidative stress, and regulation of Ca2+ homeostasis, in addition to participating in the body’s energy metabolism (108, 109). As a significant metabolic and interpreting organ, the standard structure and function of mitochondria maintain the homeostasis of the liver, and their structural abnormalities or dysfunction may become one of the pathogenic mechanisms of HCC. Apoptosis mainly includes mitochondrial pathways and membrane receptor-mediated pathways (110). In the mitochondrial pathway, the Bcl-2 family of apoptosis inhibitory molecules plays a crucial role. PUMA as an apoptosis regulator can inhibit the Bcl-2 family of anti-apoptotic molecules from promoting apoptosis (110). It has been shown that miR-518d-5p, which is aberrantly highly expressed in HCC cells, promotes sorafenib resistance by inhibiting sorafenib-induced apoptosis and, thus, sorafenib resistance (111). Mechanistically, miR-518d-5p directly targets c-Jun and downregulates the expression of PUMA, a gene downstream of c-Jun, leading to increased membrane potential and reduced ROS production. At the same time, miR-518d-5p confers a strong survival potential to HCC cells to avoid apoptosis by enhancing the buffering capacity of HCC cells against ROS and maintaining the integrity of mitochondrial membranes (111). In another study, miR-548b-3p in HCC cells was shown to inhibit the expression of the oncogenic factor CIP2A and down-regulate the expression of the CIP2A target protein c-Myc, resulting in the loss of mitochondrial membrane potential and thus triggering apoptosis (112). Interestingly, miR-548b-3p promoted cisplatin-induced apoptosis through a mitochondria-dependent pathway, thereby increasing the sensitivity of HCC to cisplatin (112). There is no doubt that miRNAs regulating mitochondrial function play a crucial role in the emergence of chemoresistance in HCC. As a complex organelle with multiple functions in the body cell, mitochondria play a pivotal role in the progression and treatment of HCC, and miRNAs regulating mitochondrial function in HCC cells can be used as potential drug resistance targets to overcome the resistance of HCC to chemotherapeutic drugs. In addition, miRNAs can also be involved in tumorigenesis and progression by regulating various cellular activities such as mitochondrial synthesis, kinetics, and metabolism. However, unfortunately, studies in this area are poorly reported and still need further exploration in the future.

## 6 Metabolic reprogramming

### 6.1 Glucose metabolism

**Figure 5:**
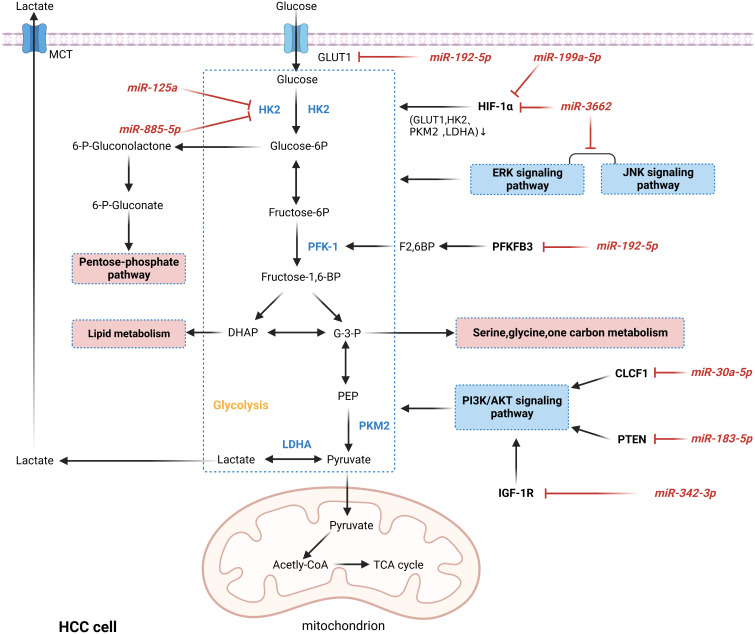
MiRNAs are involved in regulating the mechanism of aerobic glycolysis in HCC. MiRNAs act as essential regulators in the Warburg effect by down-regulating the expression levels of oncogenes or oncogenes or participating in cancer-related pathways, thereby directly or indirectly regulating critical enzymes in glycolysis and ultimately regulating the Warburg effect in HCC. In addition, intermediates in glycolysis can also enter other metabolic processes and synthesize biomolecules that contribute to adapting HCC cells to the harsh microenvironment and gain advantages in growth.

Glucose is the primary cellular energy source, primarily generated by glycolysis and mitochondrial oxidative phosphorylation pathways. Unlike normal cells, most tumor cells preferentially produce energy through the glycolytic pathway even in the presence of sufficient oxygen, as evidenced by enhanced levels of glycolysis and suppressed levels of oxidative phosphorylation, a phenomenon known as the “Warburg effect” (113). Aerobic glycolysis not only promotes the intake of glucose by tumor cells to fulfill the energy demand for rapid cell multiplication, but also its metabolic intermediates play an essential role in macromolecular biosynthesis and resistance to ROS, which gives tumor cells a certain survival advantage (114). It was confirmed that miRNAs act as essential regulators in the Warburg effect and play a crucial role in the development and progression of HCC, as shown in **Figure 5** (115–122). HIF-1α and c-Myc are the major transcription factors that promote glycolysis, and abnormal activation of ERK, JNK, and PI3K/AKT signaling pathways is also an essential mechanism of aerobic glycolysis (123, 124). It was reported that miR-3662, as a tumor suppressor of HCC, inhibited the Warburg effect and HCC growth by directly targeting HIF-1α and downregulating its expression, which regulated the expression of HIF-1α downstream target genes (GLUT1, HK2, PKM2, and LDHA) (115). Similarly, in another study, HIF-1α was inhibited by miR-199a-5p targeting, reducing glucose uptake and lactate production and suppressing the Warburg effect in HCC (122). Hexokinase 2 (HK2) catalyzes the first irreversible step in the glycolytic pathway and is a crucial player in the Warburg effect. It was shown that in hypoxic TME, miR-885-5p could directly target HK2 and downregulate its expression, inhibiting the Warburg effect in HCC (118). In addition, HK2 can be directly inhibited by miR-125a, decreasing glucose uptake and reducing lactate and ROS levels in HCC cells (119). Interestingly, the increasingly enhanced aerobic glycolysis of tumor cells creates vast quantities of lactate, leading to the accumulation and acidification of lactate in TME, further providing tumor cells with a suitable environment for survival and metastasis. For example, the deletion of miR-192-5p can upregulate the expression of two glycolytic enzymes, GLUT1 and PFKFB3 and c-Myc, which inhibits the transcription of miR-192-5p (116). Thus, the miR-192-5p/c-Myc positive feedback loop maintains excessive glycolysis in HCC cells. Furthermore, the excess lactate produced by HCC cells activates the ERK pathway in non-HCC cells, promoting cell invasiveness and stemness in HCC cells (116). Therefore, given the critical role of miRNAs in regulating glucose metabolism in HCC, targeting miRNAs to regulate the metabolic reprogramming of tumors may be a promising therapeutic approach.

### 6.2 Lipid metabolism

Lipids are mainly synthesized from fatty acids, and fatty acid anabolism in tumors can increase lipid macromolecules in large amounts, providing the necessary energy and intermediate metabolites for rapidly proliferating cells. Lipid synthesis involves several processes, among which fatty acid synthase (FASN) and stearoyl-CoA desaturase-1 (SCD1) are critical enzymes for lipid synthesis and are upregulated in rapidly proliferating cells (125, 126). It was reported that miR-4310 inhibited HCC lipid synthesis by targeting the downregulation of FASN and SCD1 expression, significantly inhibiting HCC proliferation, invasion, and metastasis *in vivo* and *in vitro* (127). Another process of fatty acid metabolism is fatty acid β-oxidation, a metabolic process in which fatty acid molecules generate acetyl coenzyme A in the mitochondria and release energy. Carnitine palmitoyltransferase 1 (CPT1) is the rate-limiting enzyme in β-oxidation of fatty acids (128). CPT1C, an isoform of the CPT1 family, translocates fatty acids into mitochondria for β-oxidation (129). Importantly, miR-377-3p has been identified as a novel inhibitor of CPT1C, capable of targeting the expression of CPT1C to reduce the rate of fatty acid β-oxidation, thereby inhibiting the growth and metastasis of HCC (129). It was shown that HADHA is a crucial factor in catalyzing the last three steps of mitochondrial β-oxidation of long-chain fatty acids. Liu et al. (130) found that HCC patients with low miR-612 expression had a poorer prognosis and shorter overall survival (OS). Further studies showed that miR-612 directly downregulates HADHA expression to regulate the fatty acid β-oxidation process, thereby inhibiting the formation of invasive pseudopods, EMT, and HCC metastasis (130). Therefore, we conclude that dysregulation of miRNAs plays a vital role in the progression of HCC by directly or indirectly affecting key enzymes or key factors in lipid synthesis or fatty acid β-oxidation processes and thus participating in lipid metabolism in HCC.

## 7 Clinical applications

### 7.1 Biomarkers

Compared with miRNAs from tissue specimens, miRNAs exist stably in serum and have the advantages of easy sampling, reproducibility, and high stability. Therefore, miRNAs have good clinical application prospects as biomarkers for both diagnosis and prognosis assessment of HCC and are rapidly becoming a hot spot for current biomarker research. HBV is a significant risk factor for HCC, and studies have shown that miRNAs can serve as potential biomarkers for HBV-associated HCC(HBV-HCC). For example, circulating miR-223-3p has good diagnostic performance (AUC=0.81, sensitivity=76.7%, specificity=80.0%) and can significantly distinguish HBV-HCC patients from healthy controls. Notably, the size of serum miR-223-3p HCC was negatively correlated with BCLC stage and could be an independent prognostic factor for the OS of HCC patients (131). In another study, serum miR-375 could distinguish between HBV-HCC patients and healthy controls (AUC = 0.838, sensitivity = 73.9%, specificity = 93.0%), which could be a valuable biomarker for HBV-HCC (132). In addition, as exosomes contain various bioactive substances, including miRNAs, it is considered to have great potential as a biomarker for HCC. Chen et al. (133) demonstrated the diagnostic value of serum Exo-miR-34a for the early diagnosis of HCC and the diagnostic potential of the combination of Exo-miR-34a and AFP for diagnosis (AUC= 0.855, sensitivity = 68.3%, specificity = 93.3%) were higher than those of the diagnosis alone and could distinguish between healthy people and HCC patients. Furthermore, the OS of HCC patients with low Exo-miR-34a expression was inferior to those with high expression. This suggests that Exo-miR-34a may be a biomarker for early diagnosis and prognostic monitoring of HCC (133). Notably, miRNAs have different diagnostic values at different clinicopathological stages of HCC. It has been shown that miR-223-3p, when combined with serum AFP, has a sensitivity of about 85% for early-stage cancer and 100% for detecting intermediate and advanced HCC when the combined assay is applied in comparison with the non-HCC group and healthy controls (131). Therefore, it is crucial to investigate the diagnostic potential of miRNAs in different pathological stages of HCC to monitor the progression of HCC. In **Table 4**, we review some recent research on miRNAs as biomarkers of HCC (46, 131, 133–139).

**Table 4:** MiRNAs as biomarkers of HCC.

MiRNA	Source	Expression	Clinical application	Diagnostic performance			Prognosis		Ref.
				AUC	Sensitivity	Specificity	Prognostic indicator	*P* value	
miR-223-3p	serum	down	diagnosis, prognostic prediction	0.81	76.7%	80.0%	OS	*P*=0.001	(131)
miR-4530	serum	up	diagnosis, prognostic prediction	0.77	72.0%	72.7%	OS	*P*=0.011	(134)
miR-4454	serum	up	diagnosis, prognostic prediction	0.74	79.0%	63.0%	OS	*P*=0.029	(134)
miR-497	serum	down	diagnosis, prognostic prediction	0.726	74.0%	66.0%	OS	*P*=0.040	(135)
miR-1246	serum	up	diagnosis, prognostic prediction	0.865	82.0%	80.0%	OS	*P*=0.040	(135)
miR-768-3p	serum	down	diagnosis, prognostic prediction	0.908	87.3%	80.0%	OS	*P*=0.006	(136)
miR-487b	serum	up	diagnosis, prognostic prediction	0.946	88.8%	90.9%	OS	*P=*0.003	(137)
miR-22	serum	down	diagnosis	0.866	89.3%	68.9%	–	–	(138)
miR-10b-5p	exosome	up	diagnosis	0.932	91.1%	75.0%	–	–	(139)
miR-215-5p	exosome	up	prognostic prediction	–	–	–	DFS	*P*=0.02	(139)
miR-34a	exosome	down	diagnosis, prognostic prediction	0.664	78.3%	51.7%	OS	*P*<0.05	(133)
miR-638	exosome	up	prognostic prediction	–	–	–	DFS	*P*=0.0197	(46)

OS, overall survival; DFS, disease free survival.

### 7.2 Adjuvant effect of MiRNAs on chemotherapy resistance

Studies have shown that systemic chemoresistance is one of the significant causes of treatment failure and relapse in patients with advanced HCC and is a major challenge in oncologic chemotherapy treatment. In the above paper, we described that miRNAs could affect chemoresistance in HCC by regulating the autophagy and iron death process of HCC cells. In fact, miRNAs can also directly regulate the expression of their target genes and thus affect chemoresistance in HCC. For example, miR-124-3p.1 was shown to not only directly target AKT2 and inhibit its expression to reduce FOXO3a phosphorylation but also directly bind SIRT1 to mediate the acetylation of FOXO3a (140). By maintaining the dephosphorylation and acetylation of FOXO3a, miR-124-3p.1 promoted the nuclear localization of FOXO3a and enhanced sorafenib-induced apoptosis, ultimately enhancing the efficacy of sorafenib against HCC in a dual-role. Notably, miRNAs can play a synergistic role by simultaneously regulating the expression of two target genes, thus enhancing HCC cells’ sensitivity to sorafenib (140). In addition, TACE is an important treatment option for many patients with intermediate to advanced HCC that is not surgically curable. However, chemoresistance is easily acquired during TACE treatment, resulting in an unsatisfactory prognosis of TACE-treated patients with advanced HCC. Studies have shown that miRNAs can inhibit the resistance of HCC cells to ADR, the first-line chemotherapeutic agent for TACE. For example, miR-125b deletion activated the HIF1α/pAKT loop, leading to HCC resistance to TACE (141). By targeting YAP1 and downregulating its expression, miR-590-5p decreased HCC chemoresistance to ADR (142). These studies reveal that miRNAs play an essential adjuvant role in reversing chemotherapeutic drug resistance in HCC cells and suggest a novel strategy combining targeted miRNAs and chemotherapy that could largely improve the prognosis of HCC patients.

### 7.3 MiRNA-based therapeutic strategies

MiRNAs have promising applications in HCC treatment. Currently, miRNA therapeutic strategies for HCC mainly include miRNA replacement therapy and miRNA-targeted therapy. MiRNA replacement therapy imports miRNA or miRNA mimics with therapeutic effects for HCC into hepatocytes through an exogenous pathway to achieve therapeutic purposes. For example, delivery of adeno-associated viral vector-mediated miR-3-3p into the liver of mice significantly slows liver tumor progression and improves survival (143). However, exogenous miRNAs may cause immune responses and toxic reactions, and the limitation of their delivery channels may affect their application. On the other hand, miRNA-targeted therapy is achieved by using miRNA inhibitors to regulate the expression levels of specific miRNAs and influence the role of miRNAs in cancer cells. Pu et al. found that the inhibitor API-1 interacts with peptidyl-prolyl cis-trans isomerase NIMA by targeting 1 (Pin1) to regulate miRNA biogenesis and inhibit HCC development, which could be a candidate for HCC treatment (144). Although miRNA therapeutic approaches for HCC are still in the experimental stage and there are still many challenges in clinical applications, with the continuous development of related technologies and research, miRNA therapy for HCC will become an essential strategy for HCC treatment in the future.

## 8 Conclusions and perspectives

As the fifth most common malignant tumor worldwide, HCC has been a difficult problem for clinicians due to its low early diagnosis rate, high recurrence and metastasis rates, and poor prognosis. MiRNAs are a class of endogenous non-coding single-stranded small molecule RNAs, and since their discovery, research on miRNAs has been a hot and difficult issue. In this review, we summarize the mechanisms by which miRNAs are involved in regulating the onset and progression of HCC by modulating TME, regulated cell death, mitochondrial function, and metabolic reprogramming. In addition, we describe the clinical applications of miRNAs as HCC biomarkers and affecting chemoresistance in HCC.

As the molecular biological mechanisms of miRNAs are further investigated in depth, miRNAs provide new ideas for future tumor therapy and have broad prospects for clinical applications in HCC. For example, exosomes, as novel mediators of intercellular signaling, carry miRNAs that play an essential role in developing HCC. Exosomes as drug carriers, targeting miRNAs for delivery into HCC cells, and rational modification of target genes to achieve therapeutic intervention will be one of the hot spots we need to study in the future. Therefore, we urgently need further to reveal the specific mechanisms of Exo-miRNAs secretion and delivery. Moreover, Exo-miRNAs have higher structural stability to protect miRNAs from degradation by ribonucleases than free nucleic acids detected by conventional liquid biopsies. There is no doubt that Exo-miRNAs can be exploited as novel biomarkers for HCC. However, the incorporation of circulating miRNAs into routine screening still requires additional clinical studies to replicate and validate the results. On the other hand, the mechanism of miRNAs regulating VM formation in HCC also gives us new insight into developing drugs for treating HCC. For example, the ideal anti-angiogenic therapy should be able to inhibit both angiogenesis and VM for better efficacy through dual action. Also, a large number of studies need to confirm whether the combination of anti-angiogenic drugs and anti-VM-forming drugs can increase the antitumor effectiveness. As mentioned above, the development and progression of HCC are dependent on tumor metabolic reprogramming, and miRNAs play a crucial role in this process. Therefore, altering the metabolic process of tumor cells by targeting miRNAs or directly limiting the specific nutritional requirements of tumor cells through dietary regulation may also serve as a novel strategy to inhibit HCC growth. Furthermore, miRNAs have been widely reported to affect the resistance of HCC to chemotherapeutic drugs by regulating autophagy, ferroptosis, and glycolytic processes. Studying the role of miRNAs in the chemotherapeutic response of HCC is significant in revealing the mechanism of drug resistance in HCC and developing individualized treatment regimens. MiRNAs can also be used as potential therapeutic targets to reverse the resistance of HCC to chemotherapeutic drugs. However, the development of tumor drug resistance results from the complex regulation of multiple genes and pathways, and its resistance mechanism varies at different stages of HCC progression. Therefore, it will be our research direction to explore further the mechanism of miRNAs in drug resistance of HCC and how to accurately use miRNAs to target and reverse drug resistance of HCC to assist tumor therapy. It is believed that as the research continues, the mechanism of mi RNAs in the development of HCC will be gradually clarified, and miRNAs will bring benefits to the early diagnosis and individualized treatment of HCC patients.

## Author contributions

JL, HB, and ZH contributed to the conception and design of the study. JL, HB and ZL searched and reviewed studies, extracted and analyzed the data, and wrote the first draft of the manuscript. MW, NL, and YX reviewed and edited the manuscript. CN, MW, and YX directed the project and contributed to discussion as well as reviewed and edited the manuscript. All authors contributed to the article and approved the submitted version.
